# αα-Hub domains and intrinsically disordered proteins: A decisive combo

**DOI:** 10.1074/jbc.REV120.012928

**Published:** 2020-12-29

**Authors:** Katrine Bugge, Lasse Staby, Edoardo Salladini, Rasmus G. Falbe-Hansen, Birthe B. Kragelund, Karen Skriver

**Affiliations:** 1 REPIN and The Linderstrøm-Lang Centre for Protein Science, Department of Biology, University of Copenhagen, Copenhagen, Denmark; 2 Structural Biology and NMR Laboratory, Department of Biology, University of Copenhagen, Copenhagen, Denmark

**Keywords:** hub, IDP, ligand binding, SLiM, context, dynamics, signaling, transcription, AD, activation domain, CBP, CREB binding protein, CCM2, cerebral cavernous malformation 2, ETO, eight-twenty-one, GO, gene ontology, HDAC, histone deacetylase, HF, histone-fold, HHD, harmonin homology domain, HID, HDAC interacting domain, ID, intrinsic structural disorder, IDR, intrinsically disordered region, ITC, isothermal titration calorimetry, MD, molecular dynamics, NCBD, nuclear coactivator binding domain, NHR1, nervy homology region 1, PAH, paired amphipathic helix, PARP, poly(ADP-ribose)polymerase, RCD1, Radical-Induced Cell Death1, RST, RCD1, SRO, and TAF4, RTEL1, regulator of telomere elongation helicase 1, SLiM, short linear motif, TAF4, transcription initiation factor TFIID-subunit 4, TAFH, TATA-box associated factor homology, TF, transcription factor, TRD, TF regulatory domain

## Abstract

Hub proteins are central nodes in protein–protein interaction networks with critical importance to all living organisms. Recently, a new group of folded hub domains, the αα-hubs, was defined based on a shared αα-hairpin supersecondary structural foundation. The members PAH, RST, TAFH, NCBD, and HHD are found in large proteins such as Sin3, RCD1, TAF4, CBP, and harmonin, which organize disordered transcriptional regulators and membrane scaffolds in interactomes of importance to human diseases and plant quality. In this review, studies of structures, functions, and complexes across the αα-hubs are described and compared to provide a unified description of the group. This analysis expands the associated molecular concepts of “one domain–one binding site”, motif-based ligand binding, and coupled folding and binding of intrinsically disordered ligands to additional concepts of importance to signal fidelity. These include context, motif reversibility, multivalency, complex heterogeneity, synergistic αα-hub:ligand folding, accessory binding sites, and supramodules. We propose that these multifaceted protein–protein interaction properties are made possible by the characteristics of the αα-hub fold, including supersite properties, dynamics, variable topologies, accessory helices, and malleability and abetted by adaptability of the disordered ligands. Critically, these features provide additional filters for specificity. With the presentations of new concepts, this review opens for new research questions addressing properties across the group, which are driven from concepts discovered in studies of the individual members. Combined, the members of the αα-hubs are ideal models for deconvoluting signal fidelity maintained by folded hubs and their interactions with intrinsically disordered ligands.

Fast and efficient regulation of complex cellular signaling pathways is mediated by highly connected protein nodes called hubs (1, 2, 3, 4, 5). These hub proteins are intimately linked to intrinsic structural disorder (ID) (1, 2, 3, 4, 5), either containing intrinsically disordered regions (IDRs) themselves (1) or being ordered proteins binding IDRs (6, 7, 8). IDRs exist in ensembles of interconverting and dynamic states, endowing them with adaptability, multivalency, and high chemical modification potential (9, 10, 11). Indeed, ID appears to be a prerequisite for fidelity in signaling pathways through exploitation of the many different mechanisms encoded in these properties (12, 13).

Recognition of IDRs in and by hubs depends on short linear motifs (SLiMs), which are stretches of 2 to 12 residues with only a few highly conserved positions (14, 15). It has been proposed that the eukaryotic SLiMome consists of up to 1 million different SLiMs (16), but SLiMs active in hub interactions have very similar features (17). Thus, it remains enigmatic how signal fidelity is orchestrated by hubs. Several folded domains present in large scaffolding proteins act as hubs, binding IDRs, typically transcription factor (TF) regulatory domains (TRDs), *via* SLiMs. These include the TAZ (18, 19), the KIX (19), the GACKIX (20), and the αα-hub domains (21). Despite belonging to different families, the domains share structural traits such as being a relatively short chain of <100 residues that folds into topologies constructed solely by α-helices (19, 21). All the domains are also part of multidomain hub proteins carrying both order and disorder (19, 21), increasing the valency of their interactions. As an additional layer of regulation, protein cofactors bring the transcriptional machinery to target genes through interactions with TFs (22) and aid in scaffolding of the transcriptional machinery (23). How specificity and regulation within the associated multicomponent complexes are controlled is far from understood.

The αα-hub domains have only recently emerged as a group of folded hub proteins (21, 24), and hence, the full potential for understanding hub proteins from studies across these similar domains has yet to be unfolded. Furthermore, understanding their role in organizing disordered transcriptional regulators and membrane protein scaffolds in interactomes of importance to human diseases and plant quality is of broad interest. This review focuses on the αα-hub domains and brings an overview and comparative analysis of their structures, functions, complexes, and mechanisms. The αα-hub domains have low sequence identity (4%–15%) (21) and are diversely involved in distinct biological functions, while still binding ligands of similar structural and chemical properties. Thus, this group of hubs constitutes a suitable model for addressing how selectivity and specificity in interactomes are controlled and how fidelity is encoded. Through specific examples, we ask how the αα-hub domains maintain fidelity and highlight concepts and open questions related to the αα-hub *modus operandi*.

## The αα-hubs

### The αα-hub domains share key fold features

**Figure 1:**
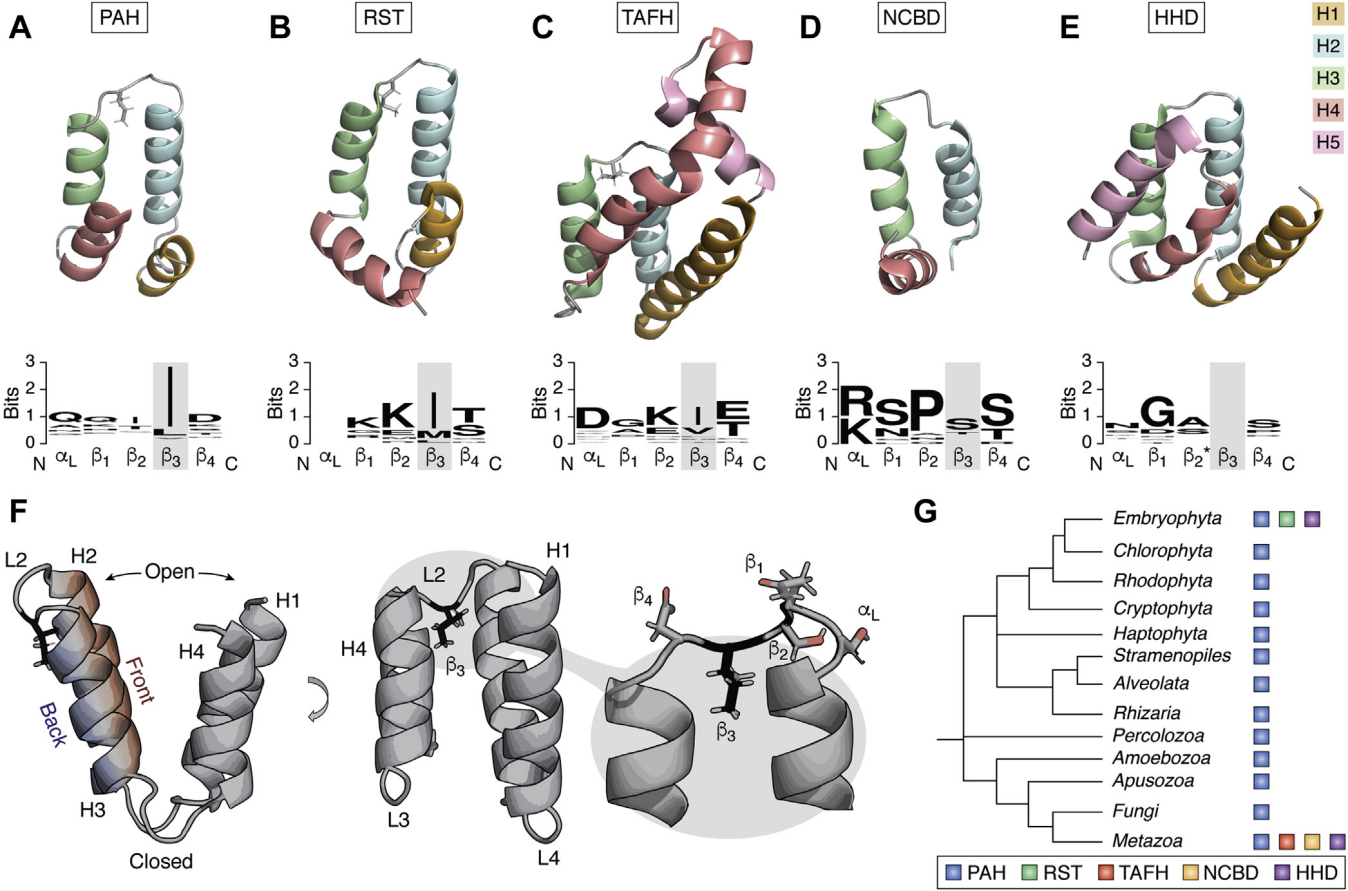
**Structures and evolution of αα-hub domains.***A*–*E*, representative structures of the current αα-hub subgroups PAH (2rmr), RST (5n9q), TAFH (2pp4), NCBD (2kkj), and HHD (4fqn), respectively. For each domain, the helices are color coded with H1 (*orange*), H2 (*blue*), H3 (*green*), H4 (*red*), and H5 (*pink*). For the domains containing the α_L_-β_4_ loop, the hydrophobic β_3_-position is shown as *gray sticks*. Sequence logos below each domain illustrate the conservation of the H2-H3 loop region across phylogenetically representative species with each position named according to the α_L_-β_4_ loop nomenclature. In the structures of HHD the β_2_-residue (marked with an *asterisk*) is located in the site normally occupied by the β_3_-residue in the α_L_-β_4_ (see also Fig. S1*C*). Empty positions indicate either lack of conservation (for RST) or the presence of a gap in the alignment (HHD). *F*, compositional features of the prototypical αα-hub. Side and front views illustrate the different surfaces, helices, and loops as defined in this review. Zoom shows the configuration of the α_L_-β_4_ loop with the hydrophobic β_3_-position forming stabilizing interactions with side chains from H2 and H3. *G*, evolutionary proliferation of αα-hubs and relationships between major eukaryotic groups (159). Branch lengths are arbitrary. *Blue*, PAH; *green*, RST; *red*, TAFH; *orange*, NCBD; *purple*, HHD.

**Figure 2:**
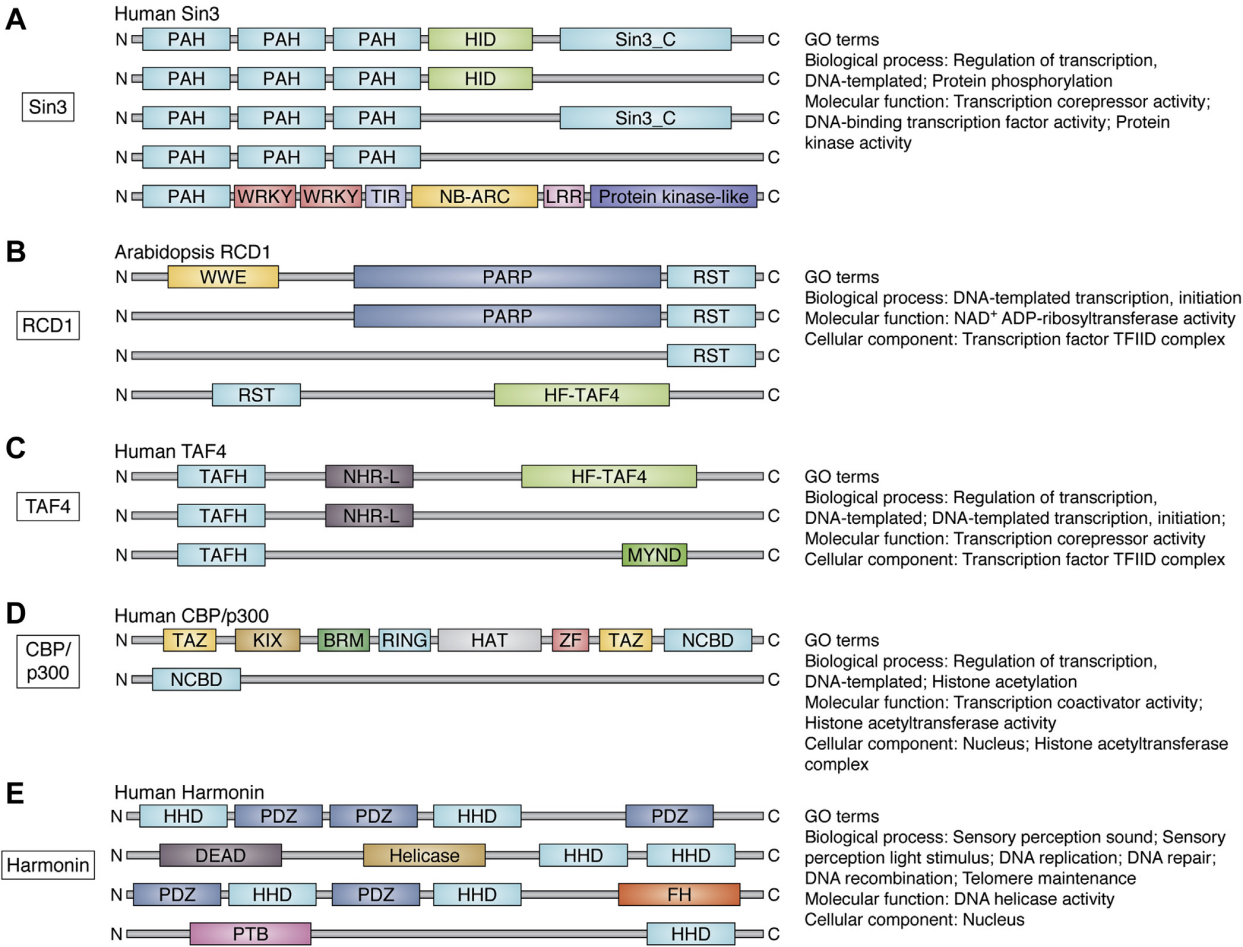
**αα-Hub protein domain structures.** αα-hub domain structures present in proteins with known functions or appearing more than 10 times in InterPro. The αα-hub domains included are: *A*, PAH, *B*, RST, *C*, TAF4, *D*, NCBD, *E*, HHD. The GO terms associated with the different αα-hub proteins are shown to the right. The schematics are not drawn to scale, the relative distance between the domains vary, and some of the proteins have more than one copy of the domains shown. GO, gene ontology.

**Figure 3:**
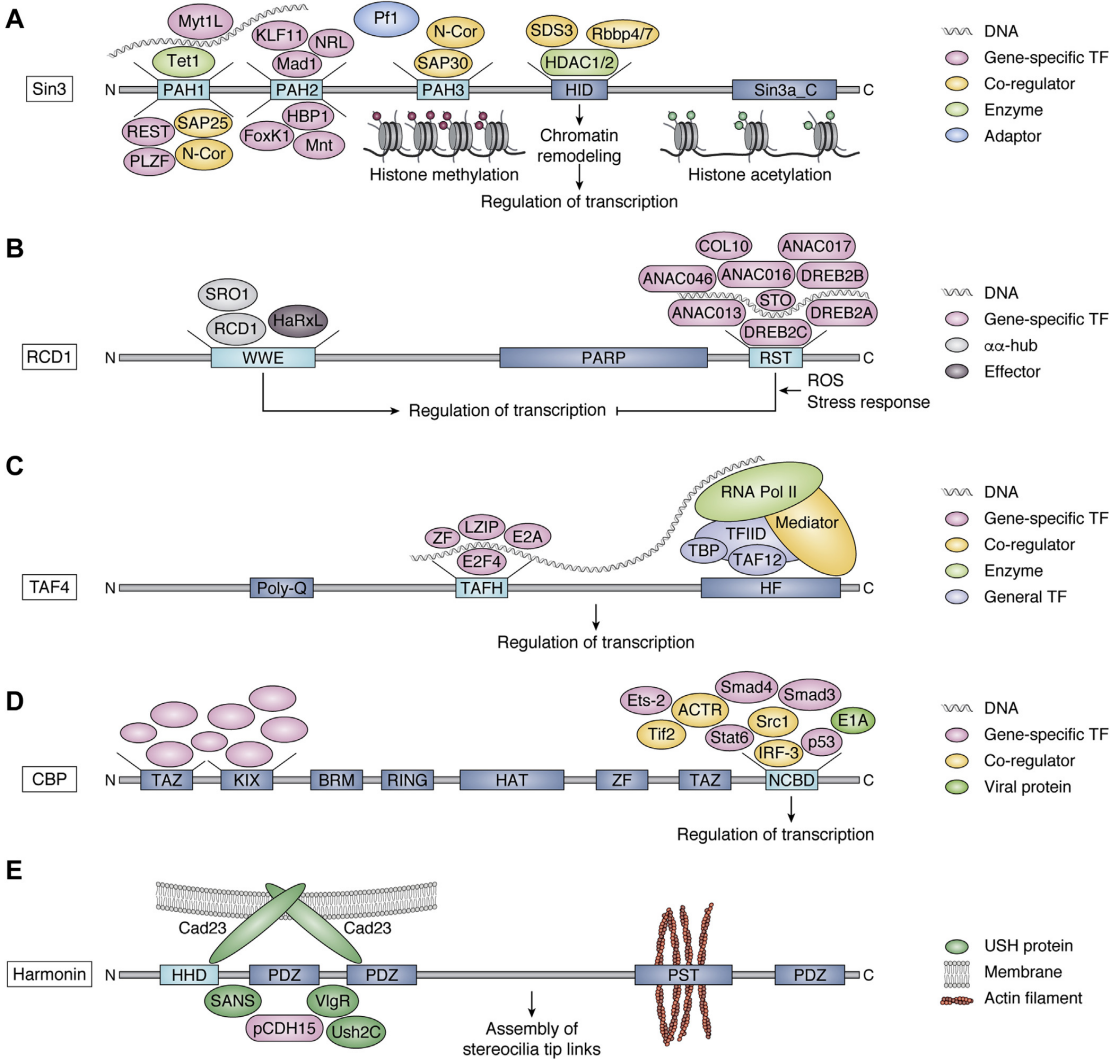
**αα-Hub protein interactions.** Examples of protein complexes and interactions of αα-hub proteins, focusing mainly on the interactions of the αα-hub domains. *A*, Sin3 as part of a coregulator complex. TFs, coregulators, adapters, and enzymes are shown bound to or as part of the binding pool of their target domains in Sin3. The classic functional role of Sin3 as a scaffold for chromatin remodeling is also shown. *B*, RCD1 in regulation of biotic and abiotic stress responses. The interactions mediate suppression of plant immunity (through HaRxL) and coordination of communication between ROS signals emitted from mitochondria and chloroplasts (through ANAC013 and ANAC017). *C*, TAF4 as part of the RNA Pol II preinitiation complex. TFs binding to the TAFH domain and implicated in embryonic pattering, and programmed cell death are shown. *D*, CBP/p300 is a central node in eukaryotic regulatory networks regulating TFs and chromatin *via* their histone acetyl transferase activity. The TAZ, KIX, and NCBD domains are scaffolds for interactions with IDRs of proteins shown with names for only NCBD ligands. *E*, Harmonin anchored interactions in regulation of hearing and vision. Cad23 assembles the upper part of the tip link, and its cytoplasmic tail is anchored to the actin filament of stereocilia *via* binding to harmonin.

The αα-hub domains were recently defined based on a common structural foundation (21) and originally included the following domains: RCD1, SRO, and TAF4 (RST) from Radical-Induced Cell Death1 (RCD1) (21); paired amphipathic helix (PAH)1, PAH2, and PAH3 from Sin3 of the Sin3/histone deacetylase corepressor complex (25); TATA-box associated factor homology (TAFH) (or nervy homology region 1 [NHR1]) from transcription initiation factor TFIID-subunit 4 (TAF4) and eight-twenty-one (ETO) (or MTG8/CBFA2T1) (26); and nuclear coactivator binding domain (NCBD) (or IRF-binding domain) from CREB binding protein (CBP) (27) (Figure 1, Figure 2, Figure 3, *A*–*D*). In addition, the harmonin homology domain (HHD) (or harmonin-N-terminal domain) from whirlin (28), harmonin (29), cerebral cavernous malformation 2 (CCM2) (30), and regulator of telomere elongation helicase 1 (RTEL1) (31) was assigned to the αα-hub domain group (24) (Figs. 1*E* and 2*E*).

The αα-hub domains consist of 3 to 5 α-helices of ∼10 to 20 residues. Their core defining feature is an αα-hairpin supersecondary structure motif (32) constituted by two consecutive antiparallel α-helices with a crossing angle close to 180°, connected by an inflexible loop (L2) (Fig. 1*F*). For all members, except NCBD, these two helices are helix 2 (H2) and 3 (H3) from the N terminus, and for clarity, helices are for all members numbered relative to these. In the prototypical member, L2 is folded into the five-residue link motif α_L_-β_4_ (33), with the β_3_-position carrying a well-sized (>100 Å^3^) hydrophobic side chain that anchors between H2 and H3 (Fig. 1*F*). For the PAH and TAFH domains β_3_ is either Ile, Leu, or Val, and for the RST domain either Ile or Met (Fig. 1, *A*–*C*). Connected to the H2-H3 core are typically two, but sometimes one or three, additional α-helices organized on the same side of the hairpin (Fig. 1*F*) (here referred to as the front). This leaves the other side of the hairpin (the back) accessible (Fig. 1*F*). The antiparallel organization of H2 and H3 orient the short, but flexible loops connecting to the preceding and proceeding α-helices at the same end of the fold, resulting in the formation of an “open” and “closed” end (Fig. 1*F*). Together, the helices support a hydrophobic binding cleft at the open end. The prototypical αα-hub is thus a domain in a modular protein consisting of four α-helices (H1–H4), of which H2 and H3 make up the αα-hairpin supersecondary structure stabilized by the hydrophobic β_3_-loop anchoring residue. The organization of H1 and H4 is the distinctive feature of each αα-hub subgroup, resulting in different angles to H2-H3 (Fig. 1, *A*–*E*). Based on this difference in topology, the five different subgroups of the αα-hubs can be defined: PAH1/2/3, RST, TAFH, NCBD, and HHD.

### Phylogenetic proliferation of the αα-hub domains

According to the literature and InterPro (34) searches, the αα-hubs are exclusive to eukaryotes (Fig. 1*G*). The PAH domain is present in most of the major clades of eukaryotes (35), whereas the RST domain has been reported in land plants including mosses and liverworts (36). HHD is present in animals and plants (28), whereas TAFH and NCBD have been found only in animals (37, 38). As orthologous genes have a higher degree of intron position conservation than nonorthologous genes (39), the structure of a gene may provide information about phylogenetic relationships. According to the RefSeq database at NCBI (40), plant RST genes and animal TAFH genes have a conserved intron position right before the α_L_-β_4_ link motif, which is missing in the remaining αα-hubs. Since RST is unique to land plants and TAFH is unique to animals, but both are present in TAF4 proteins (Figs. 2, *B*–*C* and 3C) (36, 37) and they have a conserved intron structure, an early evolutionary relationship between these domains is likely. Thus, αα-hub domain proteins are dominant in higher eukaryotes with a likely evolutionary link between TAFH and RST dividing them into two different kingdoms of life. The remaining αα-hubs have no obvious evolutionary links, except for their structural similarities.

### The αα-hub subgroups have distinctive features and bear characteristics of analogous folds

**Figure 4:**
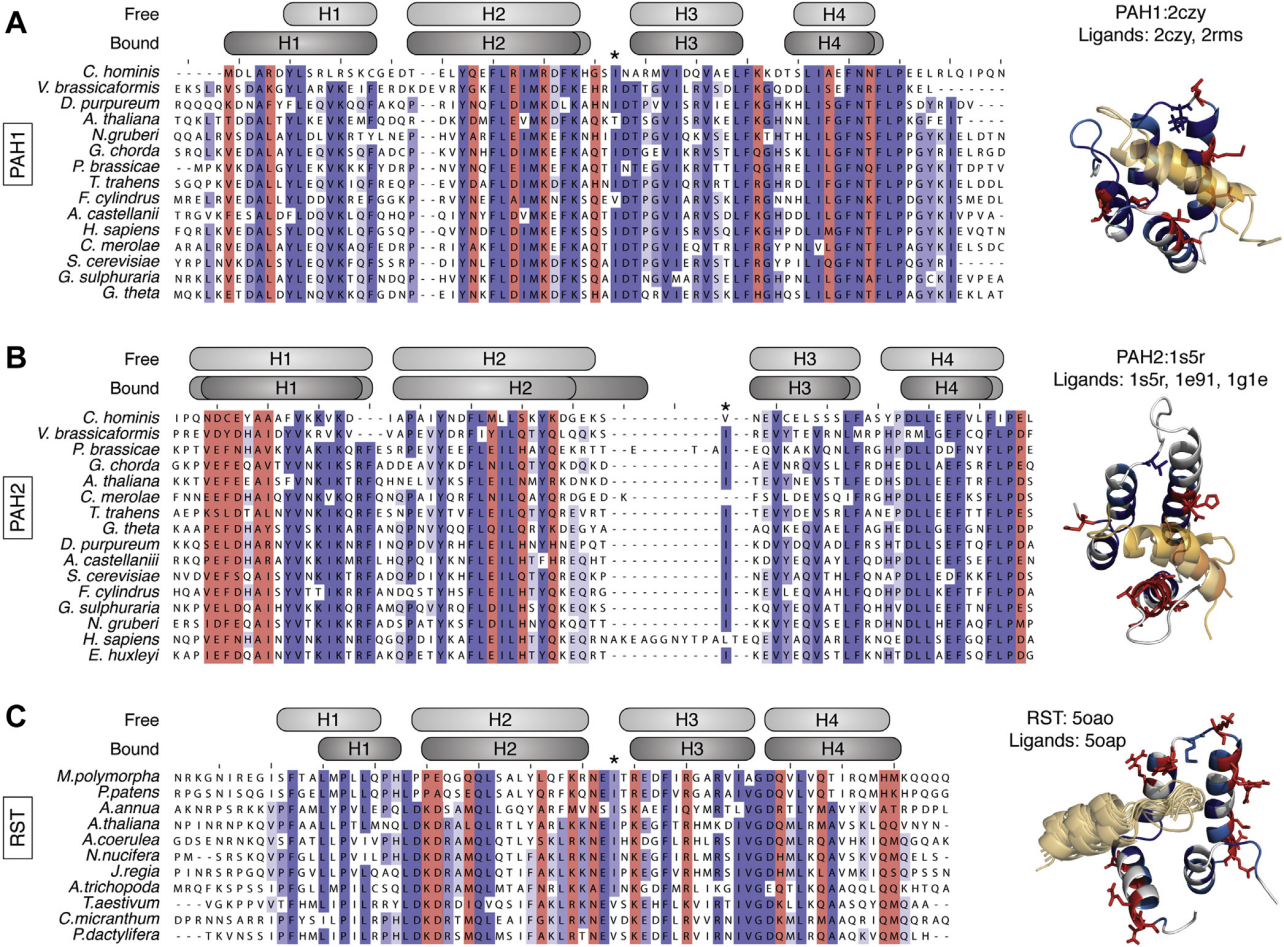
**Alignments of sequences of PAH1, PAH2, and RST, respectively, from phylogenetically representative species and comparison with 3D structures.** Sequences were aligned with Clustal Omega and visualized in Jalview. Available 3D structures of each subgroup were manually inspected and compared with the conservation alignment, and residues with identity >50% that could not be readily explained by fold-conservation (no tertiary side chain contacts) were highlighted in *red* (alignments and structures). The fold-defining positions (identity above 50% and tertiary side chain contacts) were colored *blue* in accordance with percentage identity (darker is higher identity, alignments, and structures). Above each alignment, the β_3_-position is highlighted with “∗,” and the *gray boxes* indicate the helix boundaries in the free (*light gray*) and complexed (*darker gray*, variations are different structures) αα-hubs. Species are given as four-letter abbreviations, with full names given in Table S2. *A*, PAH1. Protein Data Bank (PDB) codes 2czy, 2rms. The peptides of the ligands REST (2czy) and SAP25 (2rms) are shown semitransparent in *orange* variations. *B*, PAH2. PDB codes 1s5r, 1e91, 1g1e. The peptides of the ligands HBP1 (1s5r) and Mad1 (1e91, 1g1e) are shown semitransparent in *orange* variations. *C*, RST. PDB codes 5oao, 5oap. The ligand peptide of DREB2a (5oap) is shown semitransparent in *yellow* as an ensemble of 10 lowest-energy structures.

Proteins with similar folds, such as the αα-hub domains, can be divided into three general categories: homologs (derived from a common ancestor), remote homologs (less obvious sequential similarity because of distant ancestor), and analogs (converged to similar advantageous fold independently) (41, 42). Since the number of ways nature can arrange a few secondary structural elements in a stable manner is limited, analogous folds commonly occur for small and relatively simple protein structures (42). Furthermore, analogous structures are typically similar but with distinct features and key binding site residues (41). As alluded to above, various deviations from the prototypical features are found among the αα-hub subgroups. Some PAH2 domains differ by having extended H2s and L2s (Fig. 4*B*) (25, 43, 44, 45, 46, 47), but with persistent β_3_-anchoring (Fig. 1*A*) (21). HHD differs by generally having an atypical L2 loop lacking β_3_-anchoring, but having a H5. In CCM2-HHD, the β_2_-residue is located in the site normally occupied by the β_3_-residue and is typically a small side chain residue (Fig. 1*E* and Fig. S1*C*), while in structures of harmonin-HHD (Protein Data Bank [PDB] codes 2kbq, 2lsr, 2kbr) (48, 49), β_3_ is a Met but does not anchor between H2 and H3. For both, H5 packs between H3 and H4, possibly rescuing any lost stability from lack of β_3_-anchoring. Indeed, in harmonin-HHD the β_3_ Met interacts with side chains of H5. NCBD also lacks β_3_-anchoring, but here this coincides with a lack of H1. Of note, NCBD stands out by existing in a molten globule-like state when free (50, 51). Hence, the absence of prototypical features appears to be counteracted by helices outside the αα-hairpin, either intrinsically present in the hub or from binding partners (21). This way, the hubs may maintain stability, while exposing a hydrophobic and solvent accessible binding pocket. Taken together, the αα-hub domains have with their similar, but small, simplistic folds with distinct differences between subgroups, the typical characteristics of analogous folds.

To address conservation of the hub topology in terms of fold-defining positions across subgroups, we compared the sequences for each subgroup across phylogenetically representative species (Fig. 4, Figs. S1, and S2). 3D structures of each subgroup were manually inspected and compared with the sequence alignment to identify fold-defining positions (identity >50% and with tertiary side chain contacts). Within subgroups, many residues making up the hydrophobic core, and hence defining the fold, are highly conserved (Fig. 4, Figs. S1, and S2). Most distinctively, PAH1 and PAH2 have highly conserved cores, sharing many of the conserved core residues across all four helices, whereas PAH3 is the least conserved of all the αα-hubs (Fig. 4, *A*–*B*, and Fig. S1*A*). Across the subgroups, however, no clear conservation pattern of even H2-H3 core residues is evident, consistent with their low sequence identity (21), and despite a high degree of core residues within each subgroup. However, despite the identified evolutionary relationship described in the previous section, a sequence-relationship between RST and TAFH could not be established through this analysis (Fig. 4*C* and Fig. S1*B*). Hence, the structural similarity between the αα-hubs cannot be traced from any recognizable sequential relatedness, and besides the conserved intron structure between RST and TAFH, we found no evidence to support emergence from a common ancestor. Rather, the αα-hub folds should be considered analogous folds (52), although more extensive analysis would be required to rule out remote homology. As a consequence, the possibilities for identification of new αα-hubs directly from sequence alone is currently limited. An alternative will be searches through 3D-structure alignment using, *e.g.*, PDBeFold (53), as done in the defining work on the αα-hub group (21). However, this approach is naturally limited to targets with described 3D structures, and the identification of potential additional αα-hub group members is therefore a challenge.

## The functions of αα-hub domains

### The αα-hub domains are linked to different domains of diverse functions

To obtain an overview of the domain compositions of the αα-hub domain proteins, we searched the literature and InterPro (34). Most PAH-domain proteins, including Sin3, also contain a histone deacetylase (HDAC) interacting domain (HID) and a Sin3 C-terminal domain (Fig. 2*A*) (54), but numerous PAH-domain proteins contain only some of these domains. The PAH domain is also present in the plant protein WRKY19, which additionally contains a WRKY DNA-binding domain, a kinase domain, and a central TIR-NB-ARC-LRR module implicated in plant immunity (55). The gene ontology (GO) terms for the PAH domain proteins suggest a function in transcriptional regulation. The RST domain is found in RCD1 and is responsible for most RCD1 interactions (56, 57, 58). In addition, RCD1 contains an N-terminal WWE domain followed by a poly(ADP-ribose)polymerase (PARP) domain (Fig. 2*B*). The RST domain is also present in proteins lacking either the WWE or the PARP domain, or both, and in combination with a histone-fold (HF) domain in plant TAF4, which is reflected in GO terms related to transcription. Human TAF4 consists of a TAFH domain followed by a HF domain and is crucial for structural integrity of the TFIID complex (37, 59) (Fig. 2*C*). TAFH-domains are also found in conjunction with NHR-like domains and in ETO proteins, in which a MYND zinc finger for corepressor recruitment is also found (60). Overall, GO terms reveal a function of TAFH domain proteins in transcription. The multidomain proteins, CBP and its paralog p300 (19), both have histone acetyltransferase activity (Fig. 2*D*), as reflected in the GO terms associated with the NCBD-containing proteins suggesting functions within transcription. HHD is present in proteins with several PDZ domains, as in the case of whirlin (28) and harmonin (61) (Fig. 2*E*). In addition, HHD is found in combination with DEAD-, phosphotyrosine binding-, and formin homology domains (28). The GO terms for the HHD proteins suggest functions in sensory perception and teleomere maintenance. Thus, both similar and versatile functions and domains are linked to αα-hub domains.

### Orchestration of function from networks by αα-hubs

As hubs, the αα-hub domains serve to organize larger networks and multicomponent complexes. Sin3 proteins are coregulators of gene expression and implicated in processes such as cell cycle regulation, energy metabolism, senescence, and organ development (for recent reviews see (54, 62, 63)). Early studies showed that Sin3 is associated with HDAC1 and HDAC2 in multiprotein complexes, with its central domains, PAH3 and HID (Fig. 3*A*), interacting with the core complex components HDAC1, HDAC2, Rbbp4/7, SAP30, SAP18, and SDS3 (64, 65, 66, 67, 68). The Sin3–HDAC complex mediates histone deacetylation, which together with methylation, leads to gene repression (69), but Sin3 also interacts with the DNA demethylase Tet1 to regulate transcription epigenetically (70). The PAH1 and PAH2 domains bind numerous TFs, as shown using various different methods including biochemical methods such as pulldown assays (44, 71, 72, 73, 74, 75) and fluorescence anisotropy (76), genetic methods such as yeast two-hybrid assays (75, 77) and biophysical methods such as NMR spectroscopy (44, 76, 78). The TF ligands include REST/NRSF (71), PLZF (72), Mad1/Mdx1 (45), NRL (79), HBP1 (44), FoxK1/MNF-β (75), Mnt/Rox (73), KLF11/TIEG2 (74), and Myt1L (76), which recruit the Sin3 complex to target genes to regulate expression (54). The importance of the PAH domain:ligand interactions is apparent from several studies. For example, Tet1 depends on interactions with Sin3a-PAH1 for repression of transcription in cells (80) and for PLZF, which interacts with Sin3a-PAH1, histone deacetylation inhibition interferes with its ability to mediate transcriptional repression (72).

In agreement with RCD1 being a hub (56, 81), *rcd1 knockout* mutants, which have premature stop codons in the region encoding the PARP domain (Fig. 2*B*), thus affecting the RST and PARP domains, display pleiotropic phenotypes in gene expression, stress responses, and developmental processes. More specifically, *rcd1* shows increased ozone and salt sensitivities, changed leaf morphologies and early flowering times, as well as altered stomatal regulation (56, 81, 82, 83). The WWE domain interacts with itself and the RCD1 paralog SRO1, and with the downy mildew effector HaRxL106 to suppress plant immunity (84) (Fig. 3*B*). The interactions of RCD1-RST with TFs, which have been studied using both yeast two-hybrid assays and biophysical techniques (21, 56, 58), play important roles in plant biology. Thus, the RST domain of RCD1 affects stress responses *via* interactions with DREB2a (85) and ANAC013 and ANAC017 (86, 87), the latter two of which contribute to coordination of reactive oxygen species signals emitted from mitochondria and chloroplasts (86).

The TAFH protein TAF4 is crucial for structural integrity of the TFIID complex (59), which contains 13 additional TAF subunits and TATA binding protein (TBP) that by binding to genes triggers formation of the transcriptional preinitiation complex (37, 88, 89) (Fig. 3*C*). This, in addition, contains RNA polymerase II, general TFs, and the large Mediator complex. Through HF domains, TAF4 interacts with TAF12 to stabilize the TFIID complex (90). The TAFH domain contributes to the regulation of the expression of approximately 400 genes (91) and has been experimentally shown to interact directly with TFs such as ZF and LZIP (26). TAFH binding of the E-protein TFs, HEB and E2A, implicated in embryonic pattering and programmed cell death (92), is critical to gene activation by enhancing TFIID promoter binding (91).

CBP is a central node in eukaryotic regulatory networks (19) and regulates TFs and chromatin *via* its histone acetyl transferase activity (93). The TAZ, KIX, and NCBD domains form the scaffold for the interactions of CBP with IDRs of regulatory proteins (19). NCBD alone has multiple experimentally identified interaction partners, including IRF-3 (27), p160 nuclear receptor coactivator 1 (NCOA1;Src1), NCOA2 (Tif2), and NCOA3 (ACTR) (27, 94, 95), tumor suppressor p53 (96), Ets-2 (27), Smad3 and 4 (97), Stat6 (98), and the adenoviral protein E1A (27) (Fig. 3*D*). Also, in these cases, the ligands may depend on interactions with the αα-hub domain, both *in vivo* and *in vitro*, as in the transcriptional activation by IRF-3 (27).

The HHD proteins whirlin and harmonin are implicated in Usher syndrome causing hearing-vision loss (99). Usher syndrome proteins are organized in interactomes with harmonin, whirlin, and sans as scaffolds and cadherin23 (Cad23), protocad15, sans, VlgR, and Ush2C binding to harmonin (Fig. 3*E*) (100, 101). Structural and thermodynamic analyses have shown that HHD and PDZ1 of harmonin form a supramodule that binds sans with high affinity (48), and harmonin-HHD also binds Cad23 (61). This interaction, together with the harmonin-PDZ2:Cad23 interaction, represents multidentate binding *via* supramodule exploitation (48), providing a structural platform for the tip link complex of stereocilia (61). Furthermore, the tail of Cad23 promotes Cad23:harmonin polymer formation by binding to harmonin-HHD or by self-dimerization (49). Harmonin thus connects tip link complexes with the actin cytoskeleton (102). For an αα-hub protein, harmonin has an atypical biological function and sensory perception, but typical molecular function in scaffolding.

## The αα-hub domains as protein–protein interaction hubs

### Disordered αα-hub ligands have SLiMs of similar characteristics that maintain specificity

**Figure 5:**
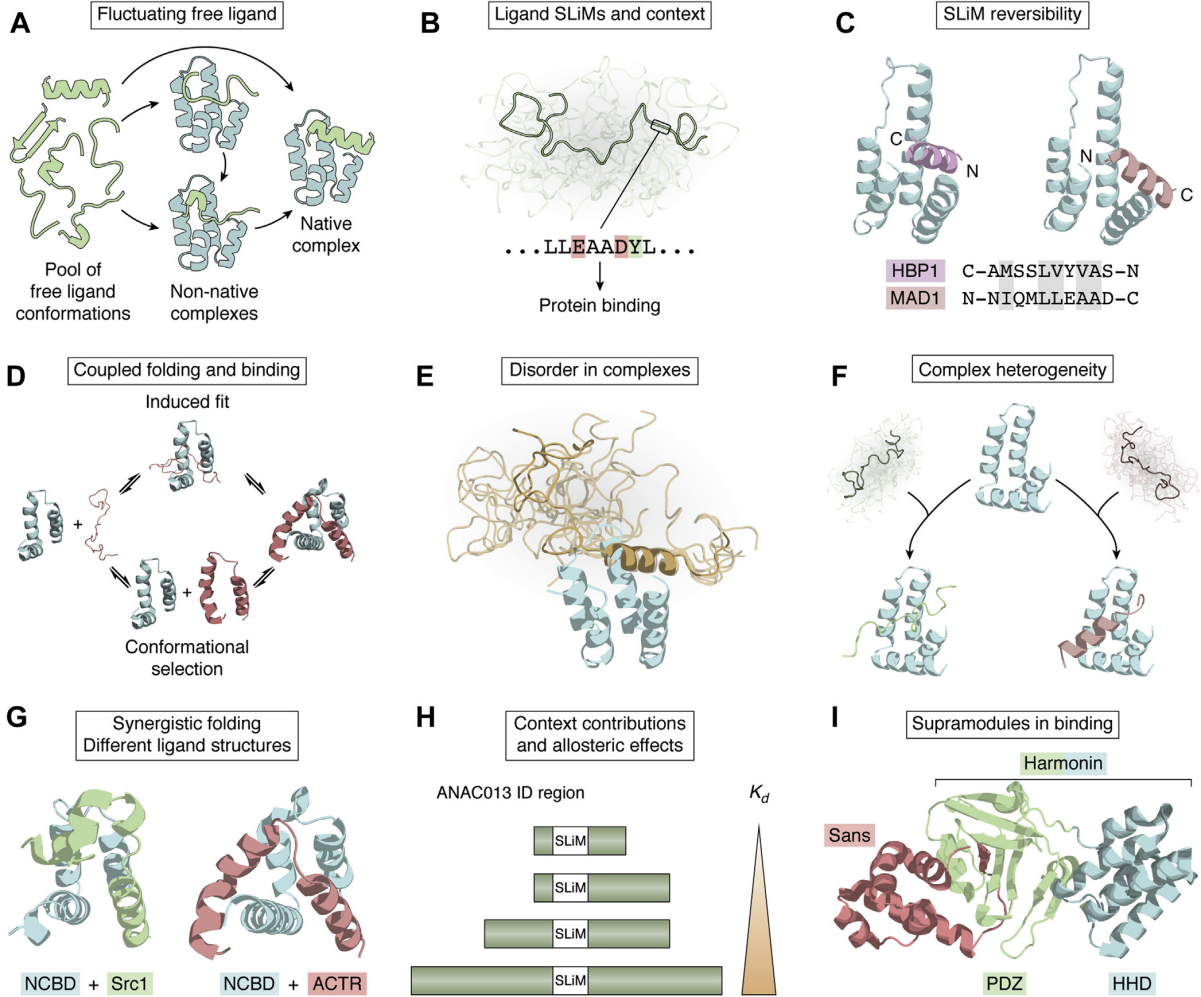
**The *modus operandi* of αα-hubs.***A*, the αα-hub-binding region of free protein ligand may fluctuate between hairpins, helices, and bent structures as in the case of the Sin3b-PAH1-binding SLiM of REST (107). *B*, protein ligand using a SLiM with hydrophobic and acidic residues for αα-hub binding as in the Sin3-PAH2-binding SLiM of Mad1 (108). The SLiM is often part of a larger intrinsically disordered context. *C*, protein ligands may use SLiM reversibility for governing specificity as in the case of Sap25 and REST binding to Sin3-PAH1 (Protein Data Bank [PDB] codes 1s5q and 1s5r) (44). *D*, ligands using coupled folding and binding, through conformational selection and/or induced fit, as in the case of ACTR association with NCBD (based on PDB codes 2kkj and 1kbh) (132). *E*, αα-Hub:ligand complexes may retain some disorder as in the case of the Sin3a-PAH1:SAP25 complex (PDB code 2rms) (103). *F*, structural heterogeneity in an αα-hub:ligand complex as in the case of RCD1-RST complexes with NAC and DREB2a transcription factors (PDB codes 5oao and 5oap) (21, 36, 58). *G*, αα-hub domains may fold synergistically with a disordered protein ligand to form different bound ligand structures as in the case of NCBD complexes with Src1 (*left*) and ACTR (*right*), respectively (PDB codes 2c52 and 1kbh) (121). *H*, allosteric effects of the SLiM context on ligand association with αα-hubs as in the case of RCD1-RST association with ANAC013 (58). *I*, αα-hubs may be part of supramodules as in the case of the harmonin:sans complex (PDB code 3k1r) (48).

Many αα-hub ligands use IDRs for binding, but identification of most αα-hub ligands dates back before the general appreciation of ID. Still, ID has often been mentioned as a feature of the free state of the hub-binding regions (46, 78, 103) or has been computationally predicted (24), whereas experimental characterization of the IDRs has mostly appeared in studies of RST (36, 57, 104, 105) and NCBD ligands (94, 96, 106). Molecular dynamics (MD) simulations have also been used for characterizing free αα-hub ligands. Thus, the Sin3b-PAH1-binding region of REST was suggested to fluctuate between hairpins, helices, and bent structures with population shifts and induced folding working cooperatively in coupled folding and binding (Fig. 5*A*) (107). ID-associated flexibility provides the structural adaptability needed for REST to function as a hub itself, and for the αα-hub ligands, ID is in general a prerequisite for adaptable SLiM-based interactions.

Although different intrinsically disordered ligands use different SLiMs for αα-hub binding, most are simple and depend on hydrophobic residues for contacts with the hydrophobic αα-hub cleft (Fig. 5*B*). Initial work to identify a PAH2-binding SLiM based on screenings, sequence comparisons, ligand affinity measurements, as well as structural analysis revealed the motif φΖΖφφΧAAΧΧφnΧΧn (X, nonproline residue; φ, bulky hydrophobic residue; Z, aliphatic side chain; n, negatively charged) (25, 43, 45, 79). Later, structural work identified two orientations of PAH-bound SLiMs, types I and II, as exemplified by the PAH1-binding SLiMs from REST (φXφφSXφS) (71, 103) and Sap25 (103) (SφXSφφXφ) (S, short side chain) (Table 1), respectively. In PAH2-complexes, the SLiMs of Pf1 and Mad1 (φΖΖφφΧAAΧΧφn) and of HBP1 (A(A/V)XφφXXφ) also adapt different orientations (Fig. 5*C*) (43, 44, 46). Despite fold similarities and SLiM simplicities, the αα-hubs show remarkable selectivity. The ∼40 times difference in affinities of Sin3-PAH2 for Mad1 (*K*_d_ ∼50 nM) (45, 103, 108) and Pf1 (*K*_d_ ∼2 μM) (46) was explained by a phenylalanine in the first position of the Pf1-SLiM constituting a steric disadvantage (46). The minimal Mad1-SLiM consists of eight residues, with only three being essential for the interactions with Sin3-PAH2. One of these, L12, inserts into the hydrophobic cleft of PAH2 and is important for affinity, whereas the other two, A15 and A16, determine specificity for PAH2, owing to their proximity to bulky side chains of PAH2 in the complex. Thus, hydrophobic residues are implicated in both affinity and specificity of PAH:SLiM interactions (108).

**Table 1:** αα-Hub-interacting SLiMs

Ligand	αα-Hub	SLiM	Reference
ACTR	CBP-NCBD	φφXXφ and φXXφφ^a^	(111)
ANAC013/016/017/046, bZIP23, COL10, DREB2a/b/c, STO	RCD1-RST	(D/E)X(1,2)(Y/F)X(1,4)(D/E)L	(58)
ANAC087	RCD1-RST	(Y/F)X(1,4)(D/E)(LI)	(36)
cMyb. HEB, N-Cor, STAT6	ETO-TAFH	(D/E)φXφφ	(109, 110)
E2A, LZIP, ZF	TAF4-TAFH	DφφXXφφ	(26)
HBP1	Sin3-PAH2	A(A/V)XφφXXφ (type II)	(44)
Mad1, Pf1	Sin3-PAH2	φΖΖφφΧAAΧΧφn (type I)	(43, 46, 108)
p53	CBP-NCBD	φφXXφ and φXXφφ	(96)
REST	Sin3-PAH1	φXφφSXφS (type I)	(71, 103)
SAP25	Sin3-PAH1	SφXSφφXφ (type II)	(103)

a X, nonproline residue; φ, bulky hydrophobic residues; Z, aliphatic component in the side chain; S, short side chain; n, negatively charged.

A combined bioinformatics and experimental approach, including substitution analysis, was used to identify the RST-binding SLiM (D/E)X(1,2)(Y/F)X(1,4)(D/E)L (where X(1,2) denotes 1 or 2 Xs) (Table 1), which has essential binding contributions from aromatic, acidic, and leucine residues (57, 58). The RST domain and the RST-binding SLiM were traced back 480 million years to the emergence of land plants, and SLiM variants, identified from the evolutionary analysis, suggested numerous additional RCD1-interactome members (36). Among the few known TAFH ligands (Fig. 3*D*), the TFs HEB, cMyb, and STAT6 and the corepressor N-Cor use the SLiM (D/E)φXφφ for binding ETO-TAFH (109, 110). Using phage display, DφφXXφφ was identified as the TAF4-TAFH-binding SLiM present in ZF, LZIP, and E2A (26). The lack of a common NCBD-binding SLiM likely reflects partner-templated modulation of the NCBD structures. However, similar SLiMs, φφXXφ or φXXφφ, mediate the interactions between NCBD and ACTR, and the TRD regions activation domain (AD)1 and AD2 of p53 (96, 111). HHDs have only a few identified ligands and no known SLiMs. Still, similar to other αα-hub ligands, hydrophobic residues are prominent in the HHD-binding ligand region (49, 61).

In summary, the simple generic φXXφφ, recurring in TF:coregulator interactions (17), is also dominant among the αα-hub-interacting SLiMs, which use both hydrophobic and charged residues for securing binding affinity and specificity. Furthermore, PAH1 and PAH2 may use SLiM reversibility for governing specificity.

### The affinities and thermodynamic profiles of αα-hub interactions vary

The affinities of the αα-hub:ligand interactions have been determined using a number of different methods including stopped-flow fluorescence spectroscopy, fluorescence titration, NMR spectroscopy, surface plasmon resonance, and isothermal titration calorimetry (ITC), with ITC being the most frequently used (43, 58, 61, 96, 106, 111, 112) (Table S1). In addition to providing information about affinities, ITC also allows determination of changes in binding enthalpy and entropy. It is generally assumed that IDRs pay an entropic cost upon binding owing to conformational restrictions (113, 114). However, IDRs may also use entropy for binding through counter-ion release (115), increased conformational flexibility (116), or expansion of the surrounding IDRs (117). For the αα-hubs, complexes form with *K*_d_s ranging from low nanomolar to mid micromolar, with most affinities in the low micromolar range (Table S1). In the high-affinity end, the Sin3a-PAH3:Sap30 complex has a *K*_d_ of 9 nM, resulting from cooperative recognition of two discrete Sin3a-PAH3 surfaces by the tripartite binding region in SAP30 (66). The high affinity may reflect constitutive Sin3:SAP30 association (68). A similar high affinity (*K*_d_ 9 nM) was measured for the RCD1-RST:ANAC013 complex, notwithstanding the lack of demonstrated induced structure in ANAC013 upon binding (58). The Sin3a-PAH2:HBP1 complex has a *K*_d_ ∼2 orders of magnitude larger than that of the Sin3a-PAH2:Mad1 complex (44, 45, 103, 108), possibly reflecting the biological functions of the two ligands with Mad1 replacing HBP1 in Sin3a complexes during differentiation (44). Large differences in affinities (40-fold) have also been detected for Sin3a-PAH1 interactions with SAP25 and Myt1L, explained by the Myt1L-SLiM diverting from the canonical SLiM (76).

The thermodynamic profiles for ligand binding vary among the hubs, even for the same αα-hub under the same experimental conditions. Some complexes are entropy driven, as exemplified by RCD1-RST complexes with Col10 (Δ*H* −9.2 kJ mol^−1^, −TΔ*S* −27.2 kJ mol^−1^; *K*_d_ 418 nM), STO (Δ*H* −3.8 kJ mol^−1^, −TΔS −36.3 kJ mol^−1^, *K*_d_ 90 nM), and ANAC087 (ΔH −15.9 kJ mol^−1^, −TΔ*S* −16.9 kJ mol^−1^, *K*_d_ 1.8 μM) (42). Other, such as the RCD1-RST:DREB2a (ΔH −63.3 kJ mol^−1^, −TΔ*S* 18.7 kJ mol^−1^, *K*_d_ 16.0 nM) and the CBP-NCBD:ACTR (Δ*H* −132.6 kJ mol^−1^, −TΔ*S* 89.1 kJ mol^−1^, *K*_d_ 34 nM) complexes are driven by enthalpy (36, 111). In a study addressing binding of Sin3 isoforms, Sin3a-PAH2 and Sin3b-PAH2 bound Pf1 with comparable affinities but apparently different thermodynamic profiles. This likely reflects that apo-Sin3a-PAH2 samples both folded and partially folded conformations and forms a monomer–dimer equilibrium and that apo-Sin3b-PAH2 is monomeric and mostly folded (see below) (43, 46, 118, 119). Accordingly, Sin3a-PAH2 and HBP1 undergo mutual coupled conformational transitions upon association (44). The thermodynamics of the αα-hub interactions with ligands thus appears diverse, ranging from highly entropically to highly enthalpically driven. However, since the different ligands can be folding to different degrees upon binding, and since both Δ*H* and *ΔS* vary with temperature, a comparison of the profiles across the different hubs is complex. Thus, it would be relevant to include more in-depth analyses under varying temperatures, which will allow determination of Δ*C*p, and through that infer on differences in binding-induced folding.

## Properties of αα-hub–ligand complexes

### The αα-hubs share a common supersite with topological variations

The majority of αα-hub complex structures have been solved with ligand peptide fragments, entailing an amphipathic α-helix bound through coupled folding and binding (Fig. 5*D*) in the hydrophobic cleft (21, 25, 43, 44, 45, 46, 71, 80, 103, 105, 120). For all the αα-hubs, this occurs without substantial changes to the backbone structure of the αα-hub, and thus while maintaining the relative helix orientations. For PAH1/2/3, RST, and HHD, binding of these ligands occurs in a shared supersite (52), consisting of the hydrophobic cleft formed at the open end of the fold (Figs. 1, *A*–*B*, *E*–*F* and 4, *A*–*C* and Fig. S1, *A* and *C*). The shared location of a binding site within apparent analogous domains suggests that it has arisen because it is a particularly advantageous structural motif (52). In this case, the open-end hydrophobic cleft seems particularly well suited for versatile binding of IDRs forming amphipathic α-helices upon binding. NCBD only fully populates the αα-hub fold upon complex formation with some ligands (111, 121) and hence does not have the supersite in a traditional sense. For PAH1/2/3 and HHD, the cleft is primarily located between H1 and H2. Here α-helices engage in a mostly hydrophobic contact surface of 650 to 750 Å^2^ (49, 61, 103), which is close to the average protein–protein interface size of 800 Å^2^ (12). For RST, the cleft opening is primarily located between H3 and H4 (21). TAFH deviates from the prototypical αα-hub traits by its hydrophobic open-end cleft (*i.e.*, the supersite) being occupied by a repositioned H4 (Fig. 1, *C* and *F* and Fig. S1*B*). Structures of TAFH complexes revealed binding of ligands in the interfaces between H1 and H4 (Fig. S1*B*), resulting in a mostly hydrophobic contact surface of 700 Å^2^ (109). This different relative orientation of H1, H4, and H5 in the αα-hubs, resulting in different positioning of side chains and geometry of the binding site, may be an additional filter for specificity tuning (21).

### The positions of binding residues are not always conserved across subgroups

The sequence alignments of the individual αα-hub subgroups presented above together with manual inspection of 3D structures allowed identification of fold-defining residues (Fig. 4, Figs. S1 and S2). However, each subgroup also revealed between 4 to 13 conserved residues that cannot be explained by apparent fold-conservation (>50% identity, lack of tertiary contacts) (Fig. 4, Figs. S1 and S2). These are likely conserved because they are crucial components of interaction sites. In, *e.g.*, PAH2, TAFH, and NCBD, 7 of 10, 6 of 7, and 10 of 13, respectively, of the suggested binding residues are in known complex structures indeed in contact with ligands (25, 43, 44, 45, 46, 96, 109, 111, 122).

In the αα-hub complex structures, the majority of ligand contacts are through the open-end hydrophobic supersite (21, 25, 43, 44, 45, 46, 48, 49, 71, 96, 103, 109, 111, 122, 123, 124). Since the analysis does not pick up residues of the hydrophobic supersite that are also part of the core fold, the identified potential binding residues are primarily solvent exposed and, hence, the majority are hydrophilic and charged (Fig. 4, Figs. S1 and S2). All the αα-hub domains have conserved binding residues in both H1 and H2 (except for H1 of RST), whereas their presence in H3, H4, and H5 varies between subgroups. The relative position of the conserved binding residues is, however, not consistently conserved throughout the domains, supporting that binding discrimination may be partially encoded in the position of key residues. Even for PAH1 and PAH2, which as described above have many common conserved core residues, the pattern of conserved binding residues is entirely different (Fig. 4, *A*–*B*). For PAH1, the 9 identified residues are distributed throughout the domain, whereas for PAH2, 6 of 10 residues are in H1 and none is in H3 and H4. Nonetheless, when inspecting their positions in available structures (Fig. 4, *A*–*B*), it is clear that they cluster around the open-end binding pocket between H1 and H2 in both PAH1 and PAH2. This difference is consistent with previous studies showing that conservative replacements of PAH2 residues with equivalent PAH1 residues were sufficient to alter affinity as well as specificity. Thus, substitution of Sin3-PAH2-Leu332, positioned in H2 of the ligand-binding cleft of PAH1/2, with Met, present in the corresponding position in PAH1, resulted in a 7-fold decrease in the affinity for Mad1 (103). For all the αα-hubs, particularly the last or second-to-last turn of H2 situated between the core and solvent-exposed side of H2 almost always has a conserved binding residue, which is in contact with ligands in known structures (Fig. 4 and Fig. S1).

NCBD is an outlier, only substantially populating the αα-hub fold with certain ligands. Here, the ligand takes the position of H1 in the complex, resulting in many conserved residues engaging in intermolecular interactions. For this reason, it is omitted from the cross comparisons. From the sequences and αα-hub-like structures of NCBD (111, 125), 13 conserved binding residues were identified, and 10 of these can be recognized as partaking in complexes (Fig. S2, (111, 125)). The remainder are on the backside of H2-H3 (two residues) or in H2 (one residue) and may engage in complexes with other ligands.

### Conserved binding residues across subgroups suggest expanded binding

Many of the identified binding-conserved residues are at the rim of the hydrophobic supersite. However, a subset has geometrically distant locations. Especially noteworthy is that all αα-hubs have conserved residues positioned at the backside of H2-H3, and a few also on the solvent-exposed side of H1 (PAH2, CCM2-HHD, RST), H4 (PAH1, RST), or H5 (CCM2-HHD). This suggests these to constitute one or more accessory binding (super)sites. A few complex structures solved with relatively large intrinsically disordered ligand fragments of ∼60 to 90 residues (Sin3-PAH1: PDB 2rms (103), Sin3-PAH3: PDB 2ld7 (66)) or folded partners (CCM2-HHD: PDB 4y5o (124), Mtgr1-TAFH: PDB 5ecj (122)) are available. In the Sin3-PAH1 complex, the additional ∼25 disordered residues do not engage with the αα-hub (Fig. 5*E*), whereas in the Sin3-PAH3 complex, the additional ∼60 residues, intrinsically disordered in the free ligand, form two α-helices engaging with conserved contact residues on the backside of H2-H3 (Fig. S1*A*). A similar pattern is observed in the CCM2–HHD complex with a folded partner (PDB 4y5o (124)), where a helix of the ligand interacts through the hydrophobic supersite, while additional contacts are made to the backside of H2-H3 (Fig. S1*C*), again including a conserved contact residue. For the complex of Mtgr1-TAFH with a larger folded partner (PDB 5ecj (122)), the αα-hub is almost completely buried within the partner, with H1 as the main anchor and the backside of H3 fully exposed (Fig. S1*B*). Hence, several structures of αα-hub complexes confirm that ligand binding is not limited to the hydrophobic supersite. This, together with the shared pattern of conserved binding residues, especially on the H2-H3 backside, suggests that αα-hub binding is more complex than a single supersite cleft, and that this is a shared property across the hubs.

### Structural heterogeneity and hub flexibility in αα-hub complexes

Protein complexes that involve IDRs may maintain varying degrees of disorder. Indeed, disorder is also preserved in some αα-hub complexes, such as the Sin3a-PAH1:SAP25 complex, in which the SAP25 peptide is largely unstructured (Fig. 5*E*) (103), although any functional implications of this remains to be determined. For complexes of RCD1-RST with different TFs, ligand plasticity is also likely. Although the α-helical structure was induced in DREB2a, no signs of helical structure were observed for ANAC046 and ANAC013 upon complex formation (Fig. 5*F*) (58). Thus, for RST different structures may form in the hydrophobic supersite, a trait so far not observed for other αα-hub interactomes.

NCBD:ligand interactions are also diverse. CBP-NCBD binds both ACTR and Src1, with the hub and the ligands existing in a molten globule-like and disordered form, respectively (111, 126), and together they cooperatively fold to form helical entities with similar CBP-NCBD folds, but different ligand (helical) topologies (Fig. 5*G*) (111, 121). CBP-NCBD and the p53-TRD also fold synergistically, with p53 forming a pair of helices docking into a hydrophobic groove of NCBD, in this case separated by a flexible chain (125).

As they have many partners (Fig. 3), αα-hub domains must maintain interdomain selectivity, and mechanisms used by other hubs may also be relevant for the αα-hubs. Here, flexibility and adaptability by the hub itself is a mechanism highly exploited by the hub’s calmodulin (127) and TAZ1 (128). However, to date, studies on the thermodynamic stability and the dynamics and malleability of the αα-hub domains remain scarce and limited by studying isolated domains. Helix dynamics of the αα-hubs from NMR- and X-ray crystallography data and thermodynamics data of their folding and unfolding (Table 2) suggests that the H2-H3 αα-hairpin is rigid and stable, whereas H1, particularly its N-terminal end, is the most dynamic part in all subgroups (21, 25, 28, 30, 44, 103). Some examples of increased dynamics in the C-terminal region of H4 also exist, primarily for αα-hubs having a fifth helix (HHD and ETO-TAFH) (28, 43) and for NCBD (126, 129), whose fold is partner dependent. For TAFH, however, it is unclear if the C-terminal dynamics has been affected by premature termination of H5. Although we cannot rule out that dynamics in the hubs arise from domain excision, the dynamics of H1 appears to be independent of the length of the N-terminal tails, and thus suggests this to be an inherent property of the fold. Some of the C-terminal regions are stabilized by folding upon binding with ligands, suggesting the formation of a localized folding transition after binding (103), whereas the lack of rigidity in the N terminus is more common after binding (25, 43, 44). Thus, the αα-hairpin super-secondary motif constitutes a structurally stable platform onto which dynamic α-helices can be organized, allowing for flexibility and subtle adaptations to binding partners.

**Table 2:** Thermodynamic parameters of the αα-hub

Domain	Δ*G* (kcal mol^−1^)	Δ*H* (kcal mol^−1^)	Δ*C*_p_ (kcal mol^−1^)	*M* (kcal mol^−1^ M^−1^)	*T*_*m*_ (°C)	Lack of rigidity^a^					Reference
						H1	H2	H3	H4	H5	
mSin3a-PAH1						X					(103)
mSin3a-PAH2						X					(44, 103)
mSin3b-PAH2						X^b^					(25, 43)
hSin3b-PAH1	12.9 ± 0.5	140 ± 1	1.53^c^		65.7 ± 0.4						(140)
hSin3b-PAH2	10.2 ± 0.5	119 ± 1	1.53 ± 0.02		64.4 ± 0.4						(140)
hSin3b-PAH3	9.9 ± 0.5	117 ± 1	1.54 ± 0.02		63.7 ± 0.4						(140)
AtRCD1-RST	3.8 ± 0.2					X					(21)
hETO-TAFH										X	(158)
hCCM2-HHD						X					(30)
whirlin-HHD					75	X				X	(28)
hCBP-NCBD	1.5 ± 0.1			0.69 ± 0.02				X			(126, 129)

a The X corresponds to lack of rigidity experimentally obtained by X-ray crystallography or NMR spectroscopy of the different ⍺-helices (H).

b The observation has been evaluated based on the structure of the complex and not on the free state.

c The value has not been experimentally determined but derived from fitting.

## Mechanisms and concepts of αα-hub *modus operandi*

### The αα-hub topology supports complex binding kinetics, cooperativity, avidity, multivalency, and supramodules

Mechanistic dissection of the αα-hub:ligand interactions is likely to contribute new conceptual understandings of hub:ID interactions, as identified in (18, 130). The interactions between CBP-NCBD and IDRs from Src1, Tif2, ACTR, and p53 showed high association rate constants (approximately 1 × 10^8^ M^−1^ s^−1^) and ionic strength dependence, reflecting the importance of electrostatics in ID-based αα-hub interactions (112). The kinetics of CBP-NCBD:ACTR association is complex, with a heterogeneous transition state reflecting an encounter complex with large structural variations (112, 131). Increased helicity in free ACTR resulted in increased *k*_on_ and decreased *k*_off_ for CBP-NCBD:ACTR (132), hinting at binding through conformational selection (Fig. 5*D*). However, MD simulations suggested that the pre-existing structure in ACTR accelerates association kinetics by promoting folding upon encounter (133). In this case, reduced *k*_off_ due to increased helicity in ACTR was explained by smaller entropic costs of forming the bound state. In another MD simulation study, CBP-NCBD was proposed to fold through global conformational selection and local induced fit upon p53 binding (134). Contributing to the complexity, two subpopulations of CBP-NCBD, *trans* and *cis*-Pro populations, bind ACTR with different affinities and kinetics, possibly representing a mechanism for interaction-based regulation of signaling (135). So far, mechanistic studies of the other αα-hubs are lacking.

Additional biochemical concepts are emerging from αα-hub:ligand studies. Thus, full-length Sin3a and a Sin3a fragment consisting of PAH1 and PAH2 interacted more strongly with Mad1 than the individual domains. PAH1 unlikely functions independently in Mad1 binding but instead cooperates with PAH2 (108). A similar pattern was seen for Sin3a:Tet1 association (80). Pf1 functions as an adaptor through multivalency by interacting with MRG15 and PAH1/2/3 and HID of Sin3 (Fig. 3*A*) (46, 136). Furthermore, two regions of Ikaros bound Sin3a and b independently (137), and N-Cor exploited two regions for interactions with Sin3b-PAH1 and −3, respectively (138). Jointly, these studies exemplify avidity in αα-hub interactions. In harmonin, the N-terminal HHD is part of a supramodule, harboring a PDZ domain and a hairpin linker, which together form a platform for strong binding of sans (Fig. 5*I*) (48). Harmonin and Cad23 interact in a tripartite manner, with two different Cad23-tail regions engaging with harmonin-HHD. The three sites of Cad23 do not display synergistic effects in harmonin binding. Instead, these multivalent interactions facilitate the formation of polymeric Cad23:harmonin complexes (Fig. 3*E*), forming a stable anchorage structure at the tip link of stereocilia (49). Together, these examples demonstrate the diversity in ways αα-hub:ligand interactions can be regulated.

### Does context matter for αα-hub interactions?

The context of αα-hub interactions is multifacial, including the disordered SLiM context of the partners, the modular protein scaffold of the hub, as well as the environment (139). However, with the exception of the PAH domains (45, 140), responses to environmental changes have not been investigated (Table 2). The Sin3b-PAH domains are sensitive to pH, being more stable at lower pH (140), where Sin3-PAH2 displays conformational heterogeneity over the pH range 4.5 to 6.0 and the temperature range 15 to 35 °C (at pH 6.0) (45). *Arabidopsis* RCD1-RST is less stable than the human Sin3b-PAH domains and has a lower melting temperature than whirlin-HHD (28). Together, this suggests that the αα-hub domains can react to different external conditions using lower stability and higher flexibility in a way that may be subgroup and species dependent. This feature could provide a correlation between regulation and environment.

For disordered proteins, both SLiM flanking regions and the remainder of the protein context are emerging as important for affinity and specificity (12, 141). For interactions such as Sin3a-PAH2 with Mad1, HBP1, and Pf1, the α-helical SLiM was regarded as the prime PAH-domain contact (44, 45, 46). However, a conserved region flanking the Pf1-SLiM may regulate competitive binding of Sin3 and MRG15 to Pf1 (46). Extension of the PAH2-binding SLiM of Mad1 with the charged region _21_RRER_24_ increased affinity ∼3-fold, suggestive of important electrostatic interactions mediated by the SLiM-flanking region (43). Furthermore, SAP30 matches the additional binding site in Sin3-PAH3 (see above) by using two α-helices in addition to the helix targeting the supersite in binding (66) (Fig. S1*A*). Thus, not only the SLiM but also flanking regions and contexts make important contributions to PAH binding.

For the RCD1-RST:TF interactions, SLiM context affected affinity as well as thermodynamic profiles. Removal of the _269_PEPEPT_274_ sequence from ANAC013(254–274) resulted in a 66-fold affinity decrease mainly from enthalpy loss, and a negative and positive allosteric effect on binding was detected for the SLiM contexts of ANAC013 (Fig. 5*H*) and DREB2a, respectively, when disordered regions outside the SLiM, which are not part of the binding site, were included in the binding experiments. For ANAC013, truncation reversed the entropic contribution to binding from negative to positive (57, 58). Thus, for the RST domain, context can be more deterministic than the SLiM itself. The isolated AD1(14–28) and AD2(38–61) regions of the p53-TRD bound CBP-NCBD approximately 180- and 8-fold weaker, respectively, than when combined in p53(1–61) (96). Similarly, the adenoviral E1A proteins, AdV5 and AdV12, contain three CBP-NCBD-binding sites, which are all required for competition with p53 in binding (106). Thus, binding is complex, likely involving allovalency (142) targeting the same site and exploiting increased local concentration effects (143). For the CBP-NCBD:ACTR association, a positive effect on binding affinity of the NCBD domain context was also demonstrated (144), highlighting the intricate regulatory potential of context, also involving the αα-hubs. The additional binding site in CCM2-HHD (see above) is reflected in bound MEKK3, where the N-terminal α-helix targeting the CCM2-HHD supersite is followed by a PB1 domain, also contributing to binding (30, 124). For harmonin-HHD binding to the Cad23 Exon68 region, the affinity decreased when Exon68 was integrated in the Cad23 protein instead of an isolated peptide, because the context of Exon68 allows for a self-dimerization, incompatible with HHD binding (49). Finally, in RTEL1, which has tandem HHDs, a PCNA interacting protein motif resides in the linker context and separates the HHDs, whereby context expands the organizational platform for the HHDs in RTEL1 (31). These examples all demonstrate that SLiM flanking regions and context play important roles also in αα-hub functionality.

In the full modular proteins, αα-hub context constitutes either two folded domains linked *via* disordered linkers or one folded domain and a disordered tail (Fig. 2). However, structural and functional studies of the αα-hubs in their intact protein contexts are lacking. The different lengths and chemical properties of the linkers and tails may effect compaction and dynamics of relevance to the accessibility of the hub (145, 146). Indeed, some of the linkers are short, which support domain cooperation as observed for PAH domains in Sin3 proteins (108), and HHD and PDZ domains in harmonin (48). Furthermore, modifications of the context, such as by phosphorylations and ubiquitylations, may enable involvement of αα-hubs in a variety of signaling processes (147). Thirteen phosphosites were recently identified for RCD1 (148), some located in the disordered N- and C-terminal contexts of the RST domain. How these affect ligand binding remains to be revealed, although not all phosphorylated residues may directly impact function.

### Why αα-hubs: open questions

The proliferation of α-helical folds in transcriptional regulation suggests this to be an optimal topology for signal integration and fidelity. In this review, we explored and compared a group of domains sharing a particular α-helical fold, the αα-hub domains, to understand why, and to pose new important questions (Table 3). The dominating model for αα-hub:ligand interactions involves paradigmatic coupled folding and binding (13, 78, 120) to form complexes of an α-helical ligand bound to an αα-hub supersite (Fig. 4). However, exceptions are emerging that suggest further advantages of this particular fold. First, SLiM contexts affect binding affinity (58) (Fig. 5*H*) and competition (106), for reasons yet to be delineated. Contexts may remain disordered in the complexes (Fig. 5*E*), and complexes may involve structural heterogeneity (Fig. 5*F*) (58), as in the Med15:Gcn4 complex (149). Avidity, of importance to the function of strong acidic ADs (150, 151), and allosteric regulation by context (Fig. 5*H*) (58) are also mechanisms of relevance to αα-hub:ligand interactions.

**Table 3:** Outstanding questions

• Is the αα-hub group complete?
• What roles do SLiM contexts play in αα-hub:ligand interactions?
• To what extent is disorder and heterogeneity present in αα-hub:ligand complexes?
• To what extent is avidity and allosteric regulation used in αα-hub:ligand interactions?
• To what extent do αα-hubs exploit more than one binding site/surface?
• Can ligand binding occur without involving the hydrophobic supersite?
• Does communication between different αα-hub-binding sites allow allosteric regulation?
• What role does αα-hub context play in selectivity?
• Is cooperativity between αα-hub domains and other (αα-hub) domains and supramodules common in binding?
• Is the structural malleability a prerequisite for binding folded partners?
• Does hub stability and flexibility determine selectivity and interactome size?
• How do posttranslational modifications affect αα-hub interactions and stability?

Second, in addition to the supersite, αα-hubs have surfaces decorated with exposed conserved residues. For many of the hubs, it remains to be established if these sites are functionally exploited and if they can be used in the absence of binding to the supersite. Furthermore, it remains to be explored if expanded and/or additional binding surfaces can impose allosteric regulation of binding, as in the case of the KIX hub domain, for which binding of one TF allosterically enhances binding of another TF to a different binding site (152, 153). The use of supramodules in αα-hub-bearing multidomain proteins (Fig. 5*I*) also points toward expandable functionality. If and how the adjoining linker contexts partake in binding and how posttranslational modifications of the hubs affect binding and selection have not yet been addressed.

Third, the H2-H3 hairpin platform combined with flexibility and dynamics of the remainder of the αα-hub topology ensures malleability and versatility in binding, also allowing binding of folded ligands. Whether stability and rigidity of the different αα-hub topologies (Table 2) correlate with specificity remains an open question. From an evolutionary point of view, higher protein–protein interaction specificity, correlating with a smaller interactome, may result in decreased network resilience, and hub domains showing malleability and promiscuity may represent an evolutionary advantage (154, 155). Comparison of ancestral and extant protein–protein interaction complexes of the plant protein SEP3 and its MAD TF ligands (156) showed that SEP3 has lost interaction partners, while increasing its structural stability (155). The fact that folded hub proteins keep flexibility as an important property is considered one of the main differences between hub and nonhub proteins (3, 157). Thus, it is possible that different degrees of flexibility, stability, and dynamics of the different αα-hubs can explain different αα-hub interactome sizes. With the uncovering of the many shared concepts valid across the hubs, and manifested by this review, it appears that the αα-hubs cope with signal fidelity and specificity using several different strategies. The similarities and differences within the hubs highlighted here establish the αα-hub domains as advantageous model systems for addressing general properties for maintaining signal fidelity in protein–protein interaction networks.

## Conflict of interest

The authors declare that they have no conflicts of interest with the contents of this article.
