# Supplementary material for: αα-Hub domains and intrinsically disordered proteins: A decisive combo
Source: J Biol Chem. 2020 Dec 29;296:100226. doi: 10.1074/jbc.REV120.012928 (PMC7948954; doi:10.1074/jbc.REV120.012928)
Supplement: Supplementary Figures and Tables [file mmc1.pdf]

Supporting Information

"αα-hub domains and intrinsically disordered proteins – a decisive combo"

[1,2]Katrine Bugge, [1,2]Lasse Staby, [1]Edoardo Salladini, [1]Rasmus G. Falbe-Hansen, [1,2*]Birthe B. Kragelund and [1,*]Karen Skriver

[1]REPIN and The Linderstrøm-Lang Centre for Protein Science

[2]Structural Biology and NMR Laboratory, Department of Biology, Ole Maaløes vej 5, 2200 Copenhagen N, Denmark

*Corresponding author: KSkriver@bio.ku.dk and bbk@bio.ku.dk

Running title: *Modus operandi* of αα-hubs

List of contents:

S-2 – S-3: Fig. S1. Alignments of sequences of PAH3, TAFH and HHD (CCM2), respectively, from phylogenetically representative species and comparison to 3D structures.

S-3: Fig. S2. Alignment of sequences of NCBD from phylogenetically representative species and comparison to 3D structures.

S-3 – S-5: Tab. S1: Affinities of αα-hub:ligand interactions.

S-5 – S-6: Tab. S2. Species abbreviations and group for sequences in Fig. 3, S1 and S2.

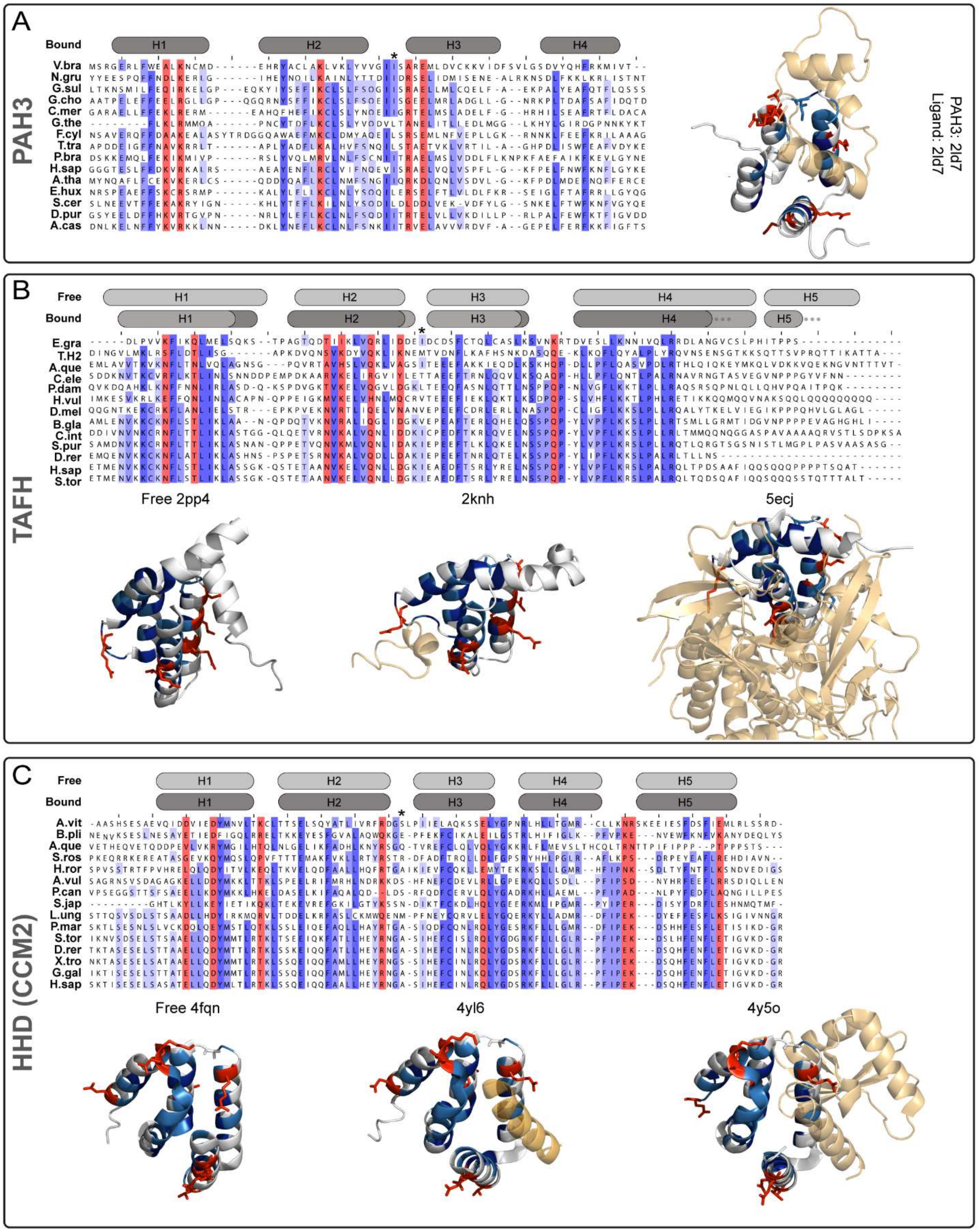

Fig. S1. Alignments of sequences of PAH3, TAFH and HHD (CCM2), respectively, from phylogenetically representative species and comparison to 3D structures. Sequences were aligned with Clustal Omega, and visualized in Jalview. Available tertiary structures of each subclass were manually inspected and compared to the conservation alignment, and residues with identity >50% that could not be readily explained by fold-conservation (no tertiary side chain contacts) were highlighted in red (alignments

and structures). The fold-defining positions (identity above 50% and tertiary side chain contacts) were colored blue in accordance with percentage identity (darker is higher identity, alignments and structures). Above each alignment, the position corresponding to the β_3-position of the α_L-β_4 loop in the structures is highlighted with "*", and the grey boxes indicate the helix boundaries in the free (light grey) and the complexed (darker grey, variations are different structures) αα-hubs. Species are given as four-letter abbreviations, with full names given in Tab. S2. **A)** PAH3. PDB code 2ld7. The SAP30 peptide ligand is shown in yellow. **B)** TAFH. PDB codes 2pp4 (free), 2knh, 5ecj. The HEB peptide (2knh) and SET domain of Prdm14 (5ecj) are shown in yellow. **C)** HHD (CCM2). PDB codes 4fqn (free), 4yl6, 4y5o. The MEKK3 peptide (4yl6) and MEKK3 (4y5o) are shown in orange and yellow.

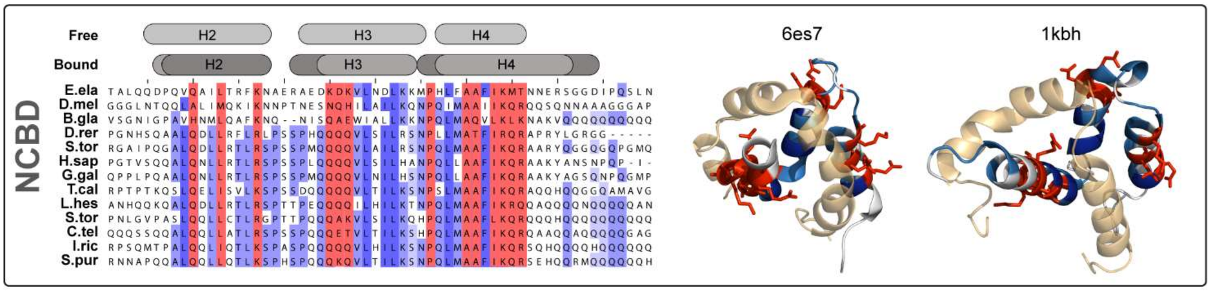

Fig. S2. Alignment of sequences of NCBD from phylogenetically representative species and comparison to 3D structures. Sequences were aligned with Clustal Omega, and visualized in Jalview. Available tertiary structures were manually inspected and compared to the conservation alignment, and residues with identity >50% that could not be readily explained by fold-conservation (no tertiary side chain contacts) were highlighted in red (alignment and structures). The fold-defining positions (identity above 50% and tertiary side chain contacts) were colored blue in accordance with percentage identity (darker is higher identity, alignment and structure). The grey boxes above the alignment indicate helix boundaries in the free (light grey) and the complexed (darker grey, variations are different structures) NCBD structures. Species are given as four-letter abbreviations, with full names given in Tab. S2. The partners NCOA3 (6es7) and ACTR (1kbh) are shown in yellow.

Table S1: Affinities of αα-hub:ligand interactions

LIGAND	αα-HUB	K_D	METHOD	REF
**ACTR(1018-1088)**	CBP-NCBD	34 nM	ITC[a]	(111)
**ACTR(1040-1080)**	CBP-NCBD	100 nM	ITC	(129)
**ACTR(1018-1088)**	CBP-NCBD	26 nM[b]	Stopped flow	(112)
**ADV5(1-36)**	CBP-NCBD	1 μM[c]	NMR	(106)
**ADV5(53-91)**		2.8 μM[c]		
**ADV12(1-32)**	CBP-NCBD	9.9 μM[c]	NMR	(106)

**ADV12(52-81)**		14 µM[c]		
**ANAC013(254–274)**	RCD1-RST	9.0 nM	ITC	(58)
**ANAC013(254-268)**		595 nM		
**ANAC013(254–299)**		32 nM		
**ANAC013(232-299)**		92 nM		
**ANAC013(161-498)**		537 nM		
**NAC016(325-367)**	RCD1-RST	200 nM	ITC	(58)
**ANAC017(296-339)**	RCD1-RST	37 nM	ITC	(58)
**ANAC046(319-338)**	RCD1-RST	609 nM	ITC	(57)
**ANAC046(264-338)**		699 nM		
**ANAC046(172-338)**		609 nM		
**ANAC087(315-335)**	RCD1-RST	1.8 µM	ITC	(36)
**BZIP23(15-36)**	RCD1-RST	128 nM	ITC	(58)
**CAD23(3181-3200)**	Harmonin-HHD	25 µM	Fluorescence titration	(61)
**CMYB(292-307)**	ETO-TAFH	21 µM	NMR	(109)
**COL10(175-208)**	RCD1-RST	418 nM	ITC	(58)
**DREB2A(255-272)**	RCD1-RST	117 nM	ITC	(58,36)
**DREB2A(244-272)**		16 nM		
**DREB2A(250-287)**		51 nM		
**DREB2A(150-335)**		27 nM		
**E2A(7-27)**	TAF4-TAFH	140 µM	ITC	(26)
**ETS-2(60-170)**	CBP-NCBD	460 nM[b]	Stopped flow	(112)
**HBP1(358-380)**	Sin3a-PAH2	5.2 µM	ITC	(44)
**HBP1(342-398)**		5.0 µM		
**HEB(11-26)**	ETO-TAFH	7 µM	ITC	(109)
**IRF-3**	CBP-NCBD	≈ 100	ITC	(112)
**LZIP(46-63)**	TAF4-TAFH	41 µM	ITC	(26)
**MAD1(9-21)**	Sin3a-PAH2	60 nM	Fluorescence polarization	(108)
**MAD1(6-21)**	Sin3a-PAH2	51 nM	Fluorescence anisotropy	(103)
**MAD1(5-20)**	Sin3b-PAH2	1.4 µM	SPR[d]	(43)
**MAD1(5-24)**		0.4 µM		
**MAD1(5-28)**		0.3 µM		
**MAD1(5-35)**		0.2 µM		
**MAD1(6-21)**	Sin3a-PAH2	29 nM	ITC	(45)
**MAD1(1-35)**		15 nM		

**MEKK3(1-124)**	CCM2-HHD	1.23 µM	ITC	(124)
**MYT1L(193-214)**	Sin3a-PAH1	6.0 µM	Fluorescence anisotropy	(76)
**N-COR(374-389)**	ETO-TAFH	75 µM	NMR	(109)
**N-COR(1829-1847)**	Sin3b-PAH1	26 µM	NMR	(71)
**P53(1-61)**	CBP-NCBD	1.7 µM	ITC and NMR	(96)
**P53(13-61)**		3.1 µM		
**P53(25-61)**		5.4 uM		
**P53(38-61)**		13.5 µM		
**P53(14-28)**		>300 µM		
**P53(13-61)**	CBP-NCBD	13 µM	ITC	(112)
**PF1(200-241)**	Sin3a-PAH2	2.2 µM	ITC	(46)
**PF1(200-241)**	Sin3b-PAH2	2.9 µM	ITC	(46)
**PF1(205-222)**	Sin3a-PAH2	580 µM	ITC	(45)
**SAP25(126-186)**	Sin3a-PAH1	134 nM	ITC	(103)
**SAP30(130-220)**	Sin3a-PAH3	9.2 nM	SPR[d]	(66)
**SRC1(920-970)**	CBP-NCBD/IBiD	12 nM[b]	Stopped flow	(112)
**STAT6(796-811)**	ETO-TAFH	> 200 µM	NMR	(109)
**STO(229-248)**	RCD1-RST	90 nM	ITC	(58)
**TET1(878-911)**	Sin3a-PAH1	<100 nM	NMR	(80)
**TIF2(1071-1110)**	CBP-NCBD	18 nM	Stopped flow	(112)
**ZF(71-90)**	TAF4-TAFH	5.2 µM	ITC	(26)

[a]ITC: isothermal titration calorimetry; [b]ionic strength = 0.074 M; [c]K_{D1}; [d]surface plasmon resonance

Table S2. Species abbreviations and group for sequences in Fig. 3, S1 and S2

Species	Abbreviation	Group
Artemisia annua	A.ann	Plant
Acanthamoeba castellanii	A.cas	Amoeba
Aquilegia coerulea	A.coe	Plant
Amphimedon queenslandica	A.que	Animal
Arabidopsis thaliana	A.tha	Plant
Amborella trichopoda	A.tri	Plant
Acanthocheilonema viteae	A.vit	Animal
Arion vulgaris	A.vul	Animal
Biomphalaria glabrata	B.gla	Animal
Brachionus plicatilis	B.pli	Animal
Caenorhabditis elegans	C.ele	Animal

Cryptosporidium hominis	C.hom	Alveolate
Ciona intestinalis	C.int	Animal
Cyanidioschyzon merolae	C.mer	Red algae
Cinnamomum micranthum	C.mic	Plant
Capitella teleta	C.tel	Animal
Drosophila melanogaster	D.mel	Animal
Dictyostelium purpureum	D.pur	Amoeba
Danio rerio	D.rer	Animal
Elaeophora elaphi	E.ela	Animal
Echinococcus granulosus	E.gra	Animal
Emiliania huxleyi	E.hux	Haptophyte
Fragilariopsis cylindrus	F.cyl	Stramenopile
Gallus gallus	G.gal	Animal
Gracilariopsis chorda	G.cho	Red algae
Galdieria sulphuraria	G.sul	Red algae
Guillardia theta	G.the	Cryptophyta
Halocynthia roretzi	H.ror	Animal
Homo sapiens	H.sap	Animal
Hydra vulgaris	H.vul	Animal
Ixodes ricinus	I.ric	Animal
Juglans regia	J.reg	Plant
Lygus hesperus	L.hes	Animal
Lingula unguis	L.ung	Animal
Marchantia polymorpha	M.pol	Plant
Naegleria gruberi	N.gru	Discoba
Nelumbo nucifera	N.nuc	Plant
Plasmodiophora brassicae	P.bra	Rhizaria
Pomacea canaliculata	P.can	Animal
Pocillopora damicornis	P.dam	Animal
Phoenix dactylifera	P.dac	Plant
Petromyzon marinus	P.mar	Animal
Physcomitrella patens	P.pat	Plant
Saccharomyces cerevisiae	S.cer	Fungus
Stichopus japonicas	S.jap	Animal
Stylophora pistillata	S.pis	Animal
Strongylocentrotus purpuratus	S.pur	Animal
Salpingoeca rosetta	S.ros	Choanoflagellate
Scyliorhinus torazame	S.tor	Animal
Triticum aestivum	T.aes	Plant
Tigriopus californicus	T.cal	Animal
Thecamonas trahens	T.tra	Apusozoa
Trichoplax sp. H2 (16S Haplotype H2)	T.H2	Animal
Vitrella brassicaformis	V.bra	Alveolate
Xenopus tropicalis	X.tro	Animal
